# Artificial intelligence assisted ultrasound for the non-invasive prediction of axillary lymph node metastasis in breast cancer

**DOI:** 10.1186/s12885-024-12619-6

**Published:** 2024-07-29

**Authors:** Xuefei Wang, Lunyiu Nie, Qingli Zhu, Zhichao Zuo, Guanmo Liu, Qiang Sun, Jidong Zhai, Jianchu Li

**Affiliations:** 1 Breast Surgery Department, Chinese Academy of Medical Sciences and Peking Union Medical College, Peking Union Medical College and Hospital, No. 3 Dongdan, Dongcheng District, Beijing, China; 2 Department of Computer Science and Technology, Tsinghua University, Beijing, China; 3 Ultrasonography Department, Chinese Academy of Medical Sciences and Peking Union Medical College, Peking Union Medical College and Hospital, No. 3 Dongdan, Dongcheng District, Beijing, China; 4 Radiology Department, Xiangtan Central Hospital, Hunan, China

**Keywords:** Breast cancer, Axillary lymph node (ALN), Sentinel lymph node (SLN), Ultrasound, Artificial intelligence

## Abstract

**Purpose:**

A practical noninvasive method is needed to identify lymph node (LN) status in breast cancer patients diagnosed with a suspicious axillary lymph node (ALN) at ultrasound but a negative clinical physical examination. To predict ALN metastasis effectively and noninvasively, we developed an artificial intelligence-assisted ultrasound system and validated it in a retrospective study.

**Methods:**

A total of 266 patients treated with sentinel LN biopsy and ALN dissection at Peking Union Medical College & Hospital(PUMCH) between the year 2017 and 2019 were assigned to training, validation and test sets (8:1:1). A deep learning model architecture named DeepLabV3 + was used together with ResNet-101 as the backbone network to create an ultrasound image segmentation diagnosis model. Subsequently, the segmented images are classified by a Convolutional Neural Network to predict ALN metastasis.

**Results:**

The area under the receiver operating characteristic curve of the model for identifying metastasis was 0.799 (95% CI: 0.514–1.000), with good end-to-end classification accuracy of 0.889 (95% CI: 0.741–1.000). Moreover, the specificity and positive predictive value of this model was 100%, providing high accuracy for clinical diagnosis.

**Conclusion:**

This model can be a direct and reliable tool for the evaluation of individual LN status. Our study focuses on predicting ALN metastasis by radiomic analysis, which can be used to guide further treatment planning in breast cancer.

**Supplementary Information:**

The online version contains supplementary material available at 10.1186/s12885-024-12619-6.

## Introduction

Breast cancer has become the leading cause of death from malignant tumor [1] according to the latest data released by the World Health Organization in 2021. The probability of death from breast cancer relates closely to metastasis, making an accurate diagnosis of axillary lymph node (ALN) metastasis crucial for staging patients. Sentinel lymph node biopsy (SLNB) has been the standard of care in assessing metastasis of breast cancer. The application of SLNB has become increasingly wide since its indication in patients with early-stage breast cancer and negative ALN, as recommended by the 2009 St. Gallen expert consensus (based on Z0011; [2]). However, not all patients are qualified for SLN resection. Candidates for SLN localization and resection should have clinically negative ALN at the time of diagnosis, or any clinically suspected ALN but negative biopsy. Moreover, SLN localization and resection must be operated by highly experienced personnel [3, 4]. However, due to limitations in SLNB training, the false negative rate (FNR) of SLNB, and complications such as lymphedema and upper limb numbness exists. Therefore, it is increasingly important to determine ALN status using alternative non-invasive methods. When treating breast cancer patients with a negative clinical physical examination but suspicious axillary lymph nodes on ultrasound (US) data, evaluation of the metastasis risk via US images can help surgeons to determine the scope of the surgical procedure when planning a sentinel biopsy. If the risk of metastasis is high, it will be more proactive to select the sentinel lymph nodes, as many uncertain factors are present in the actual operation. Blue stained lymph nodes are difficult to detect. In the absence of blue stained lymph nodes, the only options available to surgeons are the removal of enlarged lymph nodes, scintigraphy or further surgical exploration. In addition, evaluating the risk of metastasis based on US images plays an auxiliary role in the generation of a comprehensive postoperative treatment plan. If the risk of metastasis is high and the sentinel result is negative, then the adjuvant treatment approach may be more active within the recommended scope of the guide. At present, the clinical routine is to evaluate ALN through clinical pathology and breast ultrasound data. Determining the status of LNs by combining routine data from patients with new technology has become an urgent need in the field.

Ultrasound(US) has been used to evaluate ALN metastasis, especially in women with dense breasts. Research shows that the accuracy of ultrasound is higher than that of mammography and positron-emission tomography/computed tomography (PET-CT) [5–8], and that ultrasound can detect lesions that cannot be detected by mammography [6–8]. Therefore, ultrasound is widely recommended as a screening method for Asian women, since most of them are with dense breasts [8]. Although magnetic resonance imaging (MRI) and PET/CT are sensitive in predicting LN status than US, they are more time-consuming, technically complex, and expensive. Therefore, ultrasound imaging has the advantage of being portable, cost-effective, easy to operate, and applicable to ALN evaluation at levels I, II, and III compared to other imaging methods [8, 9].

Data obtained from ultrasound imaging also provide additional value in predicting the likelihood of ALN. For example, several nomograms, such as MSKCC nomogram, have been developed to predict the likelihood of SLN metastasis based on the clinicopathologic information including patient age, tumor size, tumor location, LVI(lymphovascular invasion), multifocality and histologic tumor type, etc. [10–13]. However, these nomograms and the following validation studies all failed to reach an area under the receiver operating characteristic curve (AUROC) > 0.9 [14–16]. Factors leading to such failure include subjectivity of the examiner and prediction models using logistic regression analysis. To solve this problem, some modern machine learning models have been developed. Tong et al. developed Ultrasoundbased radiomics (Statistic model) analysis for preoperative prediction of central and lateral cervical lymph node metastasis in papillary thyroid carcinoma [17]. Next, deep learning-based model called “ClymphNet” was developed by Ali Abbasian Ardakani et al. and tested in patients with papillary thyroid cancer. These two articles represent the changes in artificial intelligence methods for predicting lymph node metastasis [18]. Predictive models based on artificial neural networks (ANNs) [19, 20] and that an alternative decision tree method [21] have been established for LN estimation, both reporting good performance. We have previously established a logistic regression model that predicts LN status using ultrasound and pathological parameters [22]. However, we found substantial individual differences between sonographers in the evaluation of ultrasound focus shape, boundary, blood flow and corticomedullary boundary, etc., limiting the use of these methods. Therefore, to avoid the interference of subjective factors, we began to explore in more direct visual language learning to avoid the interference of subjective factors.

The aim of the current study is to promote individualized breast cancer care by creating a novel visual language learning model that predicts the LN status of patients who had a suspicious ALN at US, but a negative clinical physical examination.

## Materials and methods

### Ethical approval

This retrospective study was approved by the research ethics committee of Peking Union Medical College and Hospital (PUMCH). All procedures involving human participants were approved by the institutional and/or national research committee and conformed to the ethical guidelines of the Declaration of Helsinki. All participants gave written informed consent. The results were reported according to TRIPOD guidelines.

### Datasets

A total of 266 patients with invasive breast cancer were recruited at PUMCH (Peking Union Medical College & Hospital) between the year 2017 and 2019. The inclusion criteria were as follows: (a) female patients aged above 18 years with histologically confirmed staged I-III invasive breast cancer; (b) patients who had been treated with surgery and SLNB or axillary lymph node dissection (ALND), and had pathologically confirmed ALN status;

Patients were randomly assigned to training (*n* = 212), validation (*n* = 27) and test (*n* = 27) sets. Ultrasound images and pathology were analyzed in consensus by two clinicians and reviewed by a third examiner. The median follow-up was 50.5 months. Diagnosis, and treatment data were collected.

The exclusion criteria were listed below: (a) Patients who had received preoperative chemotherapy or endocrine therapy; (b) Patients with metastatic breast cancer; (c) Patients who were pregnant or lactating; (d) Patients with nipple discharge or skin diseases; (e) Patients lost to follow-up; (f) Patients with incomplete clinicopathological data.

The secretion flow was shown in Supplementary Fig. 1.

### Labels

Each evaluated ultrasound image was assigned a score based on the pathology of lymph node, as follows: 0 (no metastasis), 1 (metastasis). All results were accompanied by follow-up after diagnosis. The cases without this follow-up were excluded.

### Histopathological outcomes

Images were classified as no metastasis or metastasis in the metadata following NHSBSP guidelines. In addition, pathology reports were reviewed by board-certified pathologists and categorized according to histological findings.

Malignant pathologies included ductal carcinoma in situ, microinvasive carcinoma, invasive ductal carcinoma, invasive lobular carcinoma, special-type invasive carcinoma (tubular, mucinous, and cribriform), intraductal papillary carcinoma, non-primary BCs (lymphoma and phyllodes), inflammatory carcinoma, and phylloid tumor.

### Artificial intelligence (AI) system

**Fig. 1:**
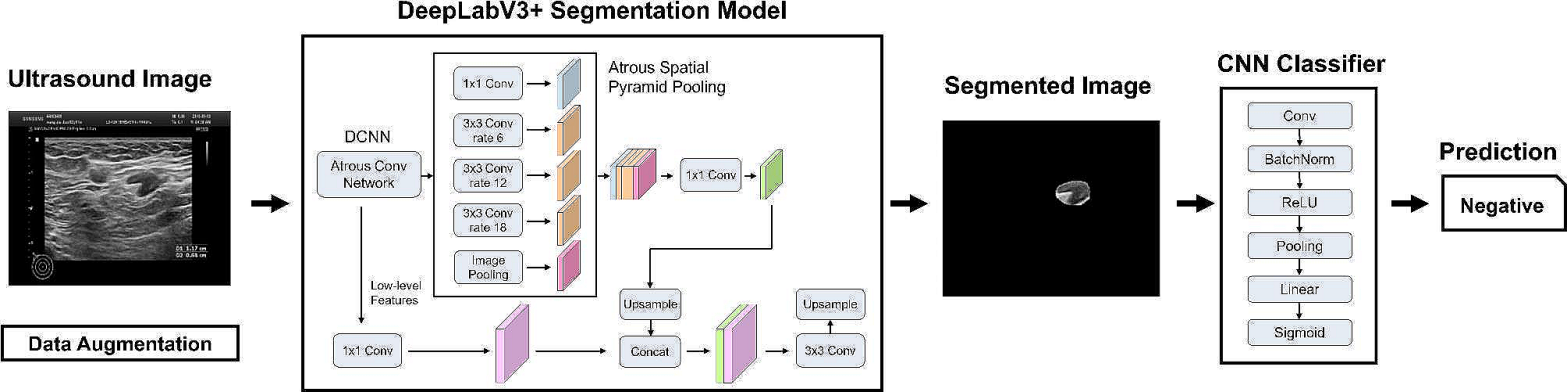
Construction principle of AI

Each patient was labeled according to pathology, as 0 (no metastasis) or 1 (more than one lymph node metastasis). As such, the problem of LN metastasis prediction was addressed as a binary classification problem. The AI system consisted of two pipeline stages, as depicted in Fig. 1. The first stage utilizes a DeepLabV3 + segmentation model [23] with ResNet-101 as the backbone network [24] that performs pixel-wise localization of the LN over the input ultrasound images. DeepLabV3 + is well-suited for this purpose as it employs atrous convolutions, also known as dilated convolutions, which allow us to capture multi-scale information effectively. This is crucial for accurately detecting lymph nodes of various sizes and shapes in ultrasound images. Subsequently, a simple Convolutional Neural Network (CNN) is implemented to perform the metastasis classification based on the segmented images.$\:\:\text{P}\text{i}\sum\:_{\text{i}=0}^{1}\text{P}\text{i}=1$ 0<$\:\text{P}\text{i}$<1. Depending on the clinical application, network parameters of the two stages are optimized together with a loss-weighted backpropagation to enhance the end-to-end performance of the models.

The convolutional neural network (CNN) used a combination of convolutional layers to automatically extract relevant features and then passed them through fully connected layers for the final prediction. Based on the post-hoc analysis, it may have used the following key features to predict axillary lymph node metastasis from segmented ultrasound images:

1. Texture Features: The network may learn to analyze the texture features of segmented lymph nodes, capturing important patterns associated with metastasis. Specifically, texture features can include information about the patterns, coarseness, and fine details within the ALN.
2. Shape Features: Shape characteristics, such as the size, irregularity, and roundness of the segmented ALN regions, were also considered during the segmentation process. Metastatic ALNs often exhibit irregular shapes, which can be a distinguishing feature for the CNN to make predictions.
3. Edge Features: Throughout the segmentation, the CNN is trained to identify boundaries and edges within the segmented ALN regions. Irregular or blurred edges may be indicative of metastasis as well.

### Image pre-processing

RGB images were captured, resized to 320 × 256 × 3, and converted into greyscale images of 320 × 256. The following augmentations were applied during the training phase using the CLoDSA Python library: random flip, random rotation between − 15° and + 15°, random translation up to 10% of image size, random pixel dropout, image sharpen, average blurring, gaussian noise, gamma correction, and elestic deformation. Then each image were normalized into a three-channel image of shape (320 × 256 × 3), then converted into a greyscale image of shape (320 × 256).

### Training

Anaconda, PyTorch, Numpy and PIL was used in feature extraction. The number of trainable parameters was approximately 22.49 million. The loss functions for the segmentation prediction and binary classification are Dice Loss, and Binary Cross Entropy, respectively. Training loss was optimized using the Adam optimizer with learning rate set to 0.0001. The neural network was trained for 100 end-to-end epochs.

### Model weight selection

The model weights that achieved maximum performance on the validation dataset were saved for test set inference. Performance was quantified using accuracy. The operating point (detection threshold) was chosen to maximize accuracy on the validation set. The operating point was derived only from the validation dataset to avoid analytical bias.

### Software

Neural network models were built using Python version 3.7.7 and the packages NumPy version 1.21.6, PIL version 8.0.1 (for image processing), Torch version 1.9.0 (for deep learning). Metric evaluation and statistical analysis were performed using Sklearn version 0.24.0.

### Statistical analysis

In this study, we evaluated the performance of the AI system using the following evaluation metrics in binary model: area under the receiver operating characteristic curve (AUROC), area under the precision-recall curve (AUPRC), sensitivity, specificity, negative predictive value (NPV), and positive predictive value (PPV). Risk scores were calculated for each patient.

Categorical variables were expressed as percentages, and statistical differences in these variables were analyzed using the chi-square test or Fisher’s exact test.

## Results

### Clinical characteristics

The clinical characteristics of our cohort are summarized in Table 1. Clinical data including age, surgery type, ultrasound, and pathology examinations were analyzed. The cohort comprised 266 patients recruited from PUMCH from January 1st 2017 to January 1st 2019, with 199 ALN^–^ (74.81%) and 67 ALN^+^ (25.19%). In a subgroup of patients specifically with ductal carcinoma in situ (DCIS) (at biopsy), 14% were ALN^+^. The median age at diagnosis was 47 years (interquartile range: 41–58 years). Patients with ALN^+^ differed significantly from those with ALN^–^ with regards to histology type (*p* = 0.043), LVI (*p* < 0.001), ALN-ultrasound corticomedullary demarcation (CMD) (*p* < 0.001), blood flow (*p* = 0.001), and progesterone receptor status (*p* = 0.009). There were no differences in nerve invasion, and infiltrative micropapillary carcinoma (IMPC) (shown in Table 1).

**Table 1:** ALN-US and basic clinical pathological characteristics of the ALN^–^ and ALN^+^ patients

			Total *N* = 266	ALN (–) *N* = 199	ALN (+) *N* = 67	*P*
Age	≤35y		31 (11.7%)	23 (11.6%)	8 (11.9%)	0.933
	>35y		235 (88.3%)	176 (88.4%)	59 (88.1%)	
Breast surgery	Mastectomy		103 (38.7%)	70 (35.2%)	33 (49.3%)	**0.041**
	Lumpectomy		163 (61.3%)	129 (64.8%)	34 (50.7%)	
ALND	Presence		63 (23.7%)	13 (6.5%)	50 (74.6%)	**< 0.001**
	Absence		203 (76.3%)	186 (93.5%)	17 (25.4%)	
ALN-US size	<1.0 cm		58 (21.8%)	42 (21.1%)	16 (23.9%)	0.634
	≥1.0 cm		208 (78.2%)	157 (78.9%)	51 (76.1%)	
ALN-US shape	Regular		260 (97.7%)	196 (98.5%)	64 (95.5%)	0.170
	Irregular		6 (2.3%)	3 (1.5%)	3 (4.5%)	
ALN-US CMD	Clear		217 (81.6%)	172 (86.4%)	45 (67.2%)	**< 0.001**
	Unclear		49 (18.4%)	27 (13.6%)	22 (32.8%)	
ALN-US blood	Absent		108 (40.6%)	92 (46.2%)	16 (23.9%)	**0.001**
	Present		158 (59.4%)	107 (53.8%)	51 (76.1%)	
Histological type ^a^	DCIS		50 (18.8%)	43 (21.6%)	7 (10.4%)	**0.043**
	IDC^b^		216 (81.2%)	156 (78.4%)	60 (89.6%)	
Tumor size	T1		166 (62.4%)	122 (61.3%)	44 (65.7%)	0.104
	T2		93 (35.0%)	74 (37.2%)	19 (28.4%)	
	T3		7 (2.6%)	3 (1.5%)	4 (6.0%)	
LVI	Presence		24 (9.0%)	10 (5.0%)	14 (20.9%)	**< 0.001**
	Absence		242 (91.0%)	189 (95.0%)	53 (79.1%)	
Nerve invasion	Presence		6 (2.3%)	4 (2.0%)	2 (3.0%)	0.644
	Absence		260 (97.7%)	195 (98.0%)	65 (97.0%)	
IMPC	Presence		8 (3.0%)	6 (3.0%)	2 (3.0%)	1.000
	Absence		258 (97.0%)	193 (97.0%)	65 (97.0%)	
ER	Positive		199 (74.8%)	144 (72.4%)	55 (82.1%)	0.113
	Negative		67 (25.2%)	55 (27.6%)	12 (17.9%)	
PR	Positive		180 (67.7%)	126 (63.3%)	54 (80.6%)	**0.009**
	Negative		86 (32.3%)	73 (36.7%)	13 (19.4%)	
Her-2	Positive		61 (22.9%)	49 (24.6%)	12 (17.9%)	0.258
	Negative		205 (77.1%)	150 (75.4%)	55 (82.1%)	
Ki67	<15%		82 (30.8%)	61 (30.7%)	21 (31.3%)	0.916
	≥15%		184 (69.2%)	138 (69.3%)	46 (68.7%)	

*Abbreviations*: ALN-US: Axillary lymph node-ultrasound; SLN: Sentinel lymph node; ALND: Axillary lymph node dissection; CMD: Corticomedullary demarcation; DCIS: Ductal carcinoma in situ; IDC: invasive ductal carcinoma; LVI: Lymphovascular invasion; IMPC: Infiltrative micropapillary carcinoma; ER: Estrogen receptor; PR: Progesterone receptor

^a^ at biopsy;^b^ including invasive ductal carcinoma, invasive lobular carcinoma and special type breast cancer

### Performance of AI system

**Fig. 2:**
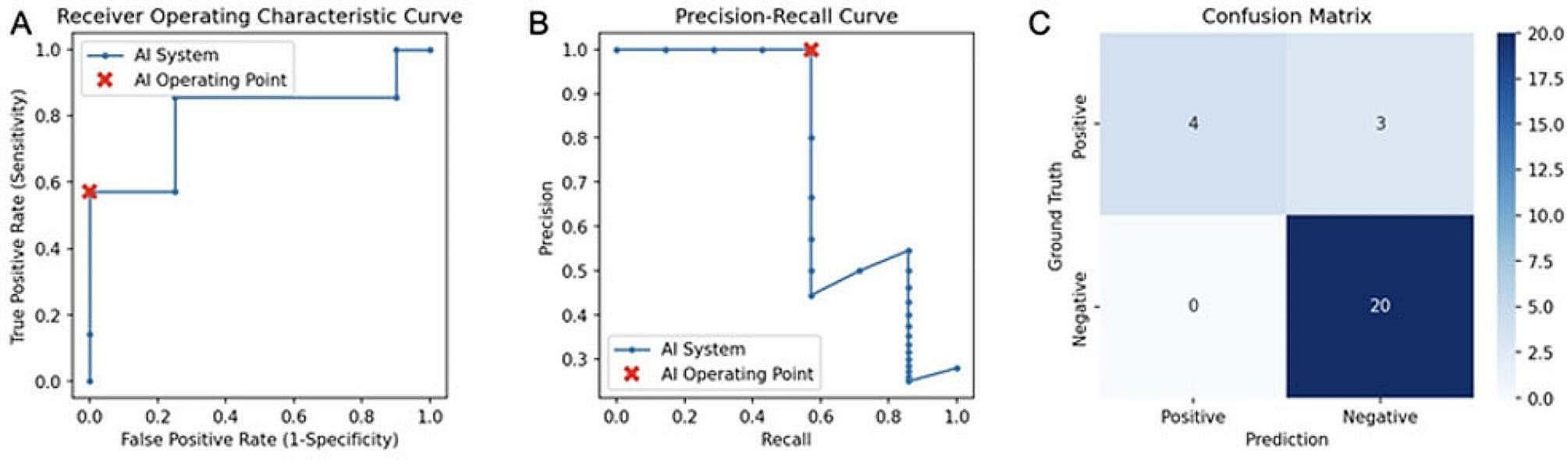
Performance of AI system(A: The area under the ROC curve was 0.799 (95% CI: 0.514–1.000) ; B: Precision recall curve of the AI system was 0.889 (95% CI: 0.741–1.000); C: Diagnostic performance of AI system and pathology using confusion matrices)

The end-to-end AI system achieved an AUROC of 0.799 (95% CI: 0.514–1.000) in identifying metastatic patients. In addition, the accuracy, sensitivity, specificity, positive predictive value (PPV), and negative predictive value (NPV) of this model were 88.89%, 57.14%, 100%, 100%, and 86.96%, respectively. The validation of the model is shown in Fig. 2. In clinical applications, this suggests that our model can be a valuable tool for clinicians in noninvasively identifying patients at higher risk of ALN metastasis.

### Potential clinical application

In view of the above results, the binary model can be used in large-scale clinical routine. The detection threshold was defined based on clinical application. In clinical practice, low-risk patients in a high-risk population are identified accurately using a low detection threshold, whereas high-risk patients in a low-risk population are identified accurately using a high threshold. We trained the model based on different operating points, and selected the model with 100% specificity and positive predictive value (PPV) for validation and determined the operating point. To avoid false negative as much as possible, the diagnostic specificity of this model for predicting breast cancer LN metastasis was 100%.

## Discussion

We developed a novel model for predicting LN metastasis based on machine learning methods. The model achieved an accuracy rate of 88.89% and a high AUROC of 79.99% and a NPV of 86.96%. To our knowledge, the performance is comparable to the highest accuracy of previously published data in the literature [5, 25, 26]. In comparison with the research that manually defined the region of interest within the LN [6], our model was based on more objective segmentation of the entire LN along with systematic subsequent analyses. Moreover, our study involved a larger sample size and consequently higher power. Our model used an non-invasive method based on artificial intelligence (AI) to assess LN metastasis. Especially for patients with breast cancer, who have a suspicious ALN on US, but a negative clinical physical examination. Its greatest advantage is that LNs can be evaluated preoperatively using only the ultrasound morphological status objectively. The practicability and value of the model are not only reflected in its convenience and rapid application, but also the decreased likelihood of false negative SLN biopsy results to some degree. Thus, in the present study, we objectively used US images to train our artificial intelligence (AI) system. This made our research results more objective.

Non-invasive AI model mainly includes non-radiomics and radiomics AI. For non-radiomics AI, which is more popular in previous years, several attempts to predict ALN status using clinical and pathological data were reported. ML(machine learning) and ANNs(artificial neural networks) are the earlier AI attempts. In 1996, Naguib et al. [27] described the earliest of these models. Marchevsky et al. [28] and Dietzek et al. [29] described similar ANNs with promising outcomes. Karakis et al. [30] employed GA-based MLP to predict ALN metastasis using nine basic clinical and pathological features. Madekiv et al. successfully established a model to help identify N2-3 LNs of early breast cancer [25].

Compared with non-radiomics, radiomics AI models can directly provide reference for diseases by acquiring more image characteristics of tumors themselves. Therefore, we developed a radiomics AI model of US that involves information automatically extracted from medical images. The earliest radiomics AI model was pioneered by Drukker et al. [6], who used US as the image source and innovated a ‘virtual biopsy’ using quantitative image analysis (QIA). They artificially selected the morphological and textural radiomics features, with the LN margin mathematically. The model achieved an AUC of 0.85 and 0.87 for metastasis prediction by node and by patient, respectively. However, a major limitation of this model and many others is identifying nodes with micro-metastasis [6]. Zarella et al. proposed a unique model that can predict ALN status using microscopic images of the primary tumor with a known ALN status. SVM classifiers were then trained and used to predict ALN status with an accuracy of 88.4% [31]. Liu et al. proposed a DCE-MRI-based radiomics model that predicts ALNM using three classifiers: SVM, XGboost, and logistic regression. SVM performed better than XGboost and logistic regression with an AUC of 0.83 and an accuracy of 0.85 [32]. SVM was also superior to KNN and LDA with 89.54% accuracy in a study conducted by Cui et al. [33]. Luo et al. also used SVM as a classifier; however, their model was unique as it utilized a group of deep features extracted and selected by CNN [34]. However, it is difficult to be applied or promoted, because that, these models pay more attention to the use of artificial intelligence methods, while ignoring the clinical validation and application. At the same time, many model parameters came from secondary processing, which still needed in application.

Indeed, there exists some parallel works that demonstrate the potential of alternative methods in improving both diagnosis and prediction. For example, using reproducible radiomics features (RFs) as utilized within a tensor fusion radiomics framework, linked with ANOVA and LR, added value to prediction of progression-free survival outcome in head and neck cancer patients [35]. Prediction of cognitive decline in parkinson’s disease using handcrafted radiomics (RF), deep (DF), and clinical (CF) features applied to hybrid machine learning systems (HMLSs) [36]. Besides Sahel repoted [37], traditional image processing techniques like Non-Local Means and morphological operations are utilized for the automatic segmentation of 3D positron emission tomography (PET) images. In prostate research [38], a U-Net convolutional neural architecture is proposed to automatically segment the prostate and its zones based on the fused images of T2W, DWI, and ADC.

Our research is a clinical application of radiation images AI visual language learning, similarly to Han et al., who applied CNN for both feature selection and classification with 84% accuracy [7]. Zhang proposed an BPNN algorithm application to achieve ultrasound image segmentation diagnosis. The segmentation accuracy reached more than 90% [39]. It is more advanced than models in previous research since this research adopts the state-of-the-art image segmentation architecture of DeepLabV3+ [23], meanwhile, analyzes the actual application scenarios of semantic segmentation of breast ultrasound images, and proposes a two-stage pipeline framework with both objectives of segmentation and classification optimized simultaneously during the loss back propagation. All the data came from our unique real-world data set. The findings from this study, reference risk value of axillary lymph node metastasis, uniquely impact on clinical decisions is mainly reflected in the assessment of model risks, assistance in selecting the surgical scope, and the selection of adjuvant treatments. Our advantage is the convenience of clinical application and the uniqueness of clinical significance. We are not proposing to replace axillary surgery, but rather to make axillary surgery more precise. We compared the relevant content [40–43] in the Supplementary Table 1.

Several limitations exist in our study as well as the literature. First, the sample size is relatively small. Since we aimed to rigorously evaluate the model performance, we intentionally kept the test set separate from the training and validation sets to prevent any data leakage during model development. We also constructed a small validation set to assess the level of data independence when selecting the model hyperparameters. We chose a specificity and positive predictive value (PPV) of 100%, which means that when our model predicts ALN metastasis, there is a high level of confidence that it is indeed present. Although this sacrificed sensitivity to some extent, we prioritized the reduction of false-positive results. Therefore, the ability to identify nodes with micro-metastasis is limited. While, the dataset size is a common limitation for AI systems, we focused on demonstrating the potential of our AI-assisted US system for the prediction of non-invasive axillary lymph node metastasis. Future work should collect additional data to address the concerns about the test and validation set sizes and further enhance the robustness and generalizability of the model. Third, to our knowledge, none of the current radiomics studies used a prospective design, and our research study is retrospective as well. Fourth, It is difficult and expensive to obtain large-scale breast ultrasound images labelled by professional doctors, which is far from meeting the needs of large-scale training data. Therefore, in our work, we adopt several image augmentation approaches to increase the amount of data annotation and alleviate the problem of overfitting during the supervised training.

However, due to the convenience of operation, our study can be applied not only to the preoperative LN evaluation of breast cancer, but also to the ALN evaluation of breast disease patients during follow-up ultrasound examination. This will greatly expand the application scope of the model. At the same time, we plan to further improve the sensitivity of lymph node prediction through the use of multi omics methods combined with artificial intelligence multimodal learning, in order to achieve the ability to identify nodes.

## Conclusion

We provide a tool that can be applied during the entire process of breast disease for predicting the LN status of patients who had a suspicious ALN at ultrasound but a negative clinical physical examination. The model achieved a validation accuracy rate of 85% and a high specificity and PPV of 100%, demonstrating high accuracy in clinical diagnosis. Prospective clinical trials with a larger population are under way.

### Electronic supplementary material

Below is the link to the electronic supplementary material.

Supplementary Material 1

Supplementary Material 2

## Data Availability

The data are not publicly available due to their containing information that could compromise the privacy of research participants.
