# Supplementary figures and images for: Artificial intelligence assisted ultrasound for the non-invasive prediction of axillary lymph node metastasis in breast cancer
Source: BMC Cancer. 2024 Jul 29;24:910. doi: 10.1186/s12885-024-12619-6 (PMC11285453; doi:10.1186/s12885-024-12619-6)

Figure legends

Supplementary Figure 1: Flowchart of this study.

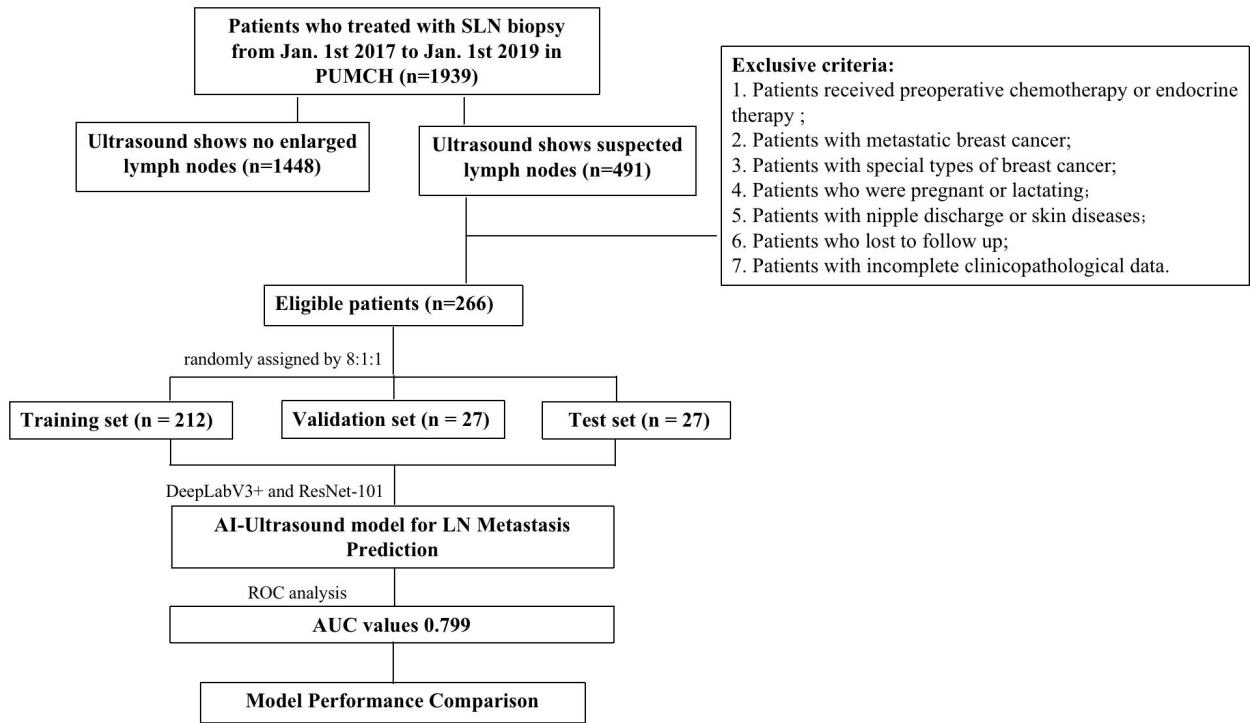

Supplement: Supplementary file 1 — Supplementary Material 1 [file 12885_2024_12619_MOESM1_ESM.pdf]
