# Supplementary material for: Artificial intelligence assisted ultrasound for the non-invasive prediction of axillary lymph node metastasis in breast cancer
Source: BMC Cancer. 2024 Jul 29;24:910. doi: 10.1186/s12885-024-12619-6 (PMC11285453; doi:10.1186/s12885-024-12619-6)
Supplement: Supplementary file 2 — Supplementary Material 2 [file 12885_2024_12619_MOESM2_ESM.docx]

**Supplementary table 1:** A literature review that summarize the representative results of previous studies focus on AI models for breast cancer LN metastasis prediction.

| **Author** | **Imaging Modality** | **Number of Patients** | **Methods** | **AUC** | **Summary/Comments** | **Year** |
| --- | --- | --- | --- | --- | --- | --- |
| Dietzel M, et al.[30] | MRI | 194 | ANN | 0.74 | ANNs for the prediction of lymph node metastases in breast MRI is feasible | 2010 |
| Drukker K, et al.[27] | Ultrasound | 90 | Selforganizing Mathematical  model | 0.85 | Mathematical descriptors of sonographically imaged lymph nodes may be useful as prognostic biomarkers | 2015 |
| Liu C, et al. [33] | DCE-MRI | 163 | LASSO | 0.806 | Potentially benefit those with negative SLN, by eliminating unnecessary invasive lymph node removal | 2018 |
| Luo J, et al. [35] | MRI | 172 | CNN+SVM+BOF | 0.852 | Noninvasive approach for automatically predicting prediction of SLN metastasis in patients with breast cancer. | 2018 |
| Cui X, et al. [34] | DCE-MRI | 102 | SVM | 0.861 | Progress in methodology | 2019 |
| Guo X, et al.[42] | Ultrasound | 937 | DLRU Pretrained ImageNet and Deep Learning Radiomics | 0.812 | May lead to a reduction in morbidities of ALND or SLND without adverse impact on survival | 2020 |
| Zhou LQ, et al [43] | Ultrasound | 756 | CNN model, Inception V3 | 0.89 | May provide an early diagnostic strategy for lymph node metastasis | 2020 |
| Zhang L, et al. [41] | Ultrasound | 90 | BPNN | / | The BPNN artificial intelligence algorithm | 2021 |
| Li Z, et al.[45] | FDG-PET/  CT | 407 | 3D residual  CNN with  attention  module | 0.868 | Even if the diagnostic performance of AI is not better than that of clinicians, taking AI diagnoses into consideration may positively impact the overall diagnostic accuracy | 2021 |
| Sun S, et al.[44] | Ultrasound | 169 | CNN with  Adam  Optimizer  training | 0.72 | It’s feasible to predict the likelihood of axillary lymph node metastasis from US images using a deep learning technique | 2022 |
| **Our study** | **Ultrasound** | **266** | **DeepLabV3+ +ResNet-101** | **0.799** | **We divide patients into low-risk and high-risk groups, and it is crucial to make decisions on the scope of surgery and adjuvant treatment** | **2023** |
